# Enhancing polygenic risk prediction by modeling quantile-specific genetic effects

**DOI:** 10.1038/s41598-026-47082-9

**Published:** 2026-04-06

**Authors:** Suin Kim, Taewan Goo, Taesung Park, Mira Park

**Affiliations:** 1 Department of Statistics, Korea University, Seoul, Republic of Korea; 2 Interdisciplinary Program in Bioinformatics, Seoul National University, Seoul, Republic of Korea; 3 Department of Statistics, Seoul National University, Seoul, Republic of Korea; 4 Department of Preventive Medicine, Eulji University, Daejeon, Republic of Korea

**Keywords:** Distributional heterogeneity, Genetic prediction, Genome-wide association study, Polygenic risk score, Quantile regression, Computational biology and bioinformatics, Diseases, Genetics

## Abstract

Polygenic risk scores (PRSs) quantify an individual’s genetic susceptibility to complex traits and diseases. Conventional PRSs, which are based on linear models, perform poorly for phenotypes with skewed distributions or with genetic effects that vary across the distribution. We propose a quantile regression-based PRS (QPRS) that can capture quantile-specific genetic effects. While existing PRSs provide only a single score, QPRS models genetic influences at multiple quantiles of the phenotype, thereby enhancing predictive performance by utilizing these multiple scores as covariates. We evaluate the performance of our method through both simulations and a real-data application. In simulations, QPRS significantly reduces the mean squared error compared to the linear-based PRS, both in the presence of variance quantitative trait loci and outliers. For real data analysis, we use data from Korea Genome and Epidemiology Study to evaluate predictive performance. We consider two prediction tasks: continuous outcomes (triglycerides and glucose level) and a binary outcome (diabetes status, derived from glucose level). QPRS demonstrates consistent improvements over conventional mean-based PRSs across both prediction tasks.

**Supplementary Information:**

The online version contains supplementary material available at 10.1038/s41598-026-47082-9.

## Introduction

Polygenic risk scores (PRSs) are a widely used tool for translating genome-wide association study (GWAS) findings into individual-level predictions of complex traits and disease risk. By aggregating genome-wide variant effects, particularly those of single-nucleotide polymorphisms (SNPs), PRSs can help identify individuals at elevated risk, guide preventive strategies, and contribute to personalized medicine^1–3^. A variety of statistical approaches exist for PRS construction, including clumping-and-thresholding, lassosum^4^, LDpred^5^, SBLUP^6^, and PRS-CS^7^. These methods differ in how they model linkage disequilibrium (LD), apply shrinkage priors, and implement penalized regression. They have been applied in various contexts, including predicting body mass index and obesity outcomes using lassosum^8^, analyzing a range of complex traits in the UK Biobank^9^, and assessing the transferability of PRS estimates for height and BMI across populations^10^.

Despite these advances, most PRS construction methods rely on GWAS based on ordinary least squares regression or related linear models, which focus on conditional mean effects^11^. This mean-centric perspective can mask heterogeneity when genetic effects vary across the phenotype distribution. For example, some variants may exert a stronger influence on the distribution tails rather than the center, yet such distributional differences are overlooked in mean-based models. Several studies have explored heterogeneity in genetic effects across trait distributions, reporting evidence that genetic influences may differ substantially depending on the quantile of the phenotype considered^12–15^.

Quantile regression (QR)^16,17^ offers a complementary perspective by estimating the genetic effects across conditional quantiles of the phenotype distribution. This approach provides a distribution-aware view of genetic associations that conventional mean-based models may not capture. QR has been used in estimating genetic effect to flowering-time traits in common bean^18^, to body mass index^12^, and other 28 continuous quantitative traits including blood glucose, total cholesterol, BMI^19^. Recently, Wang, et al. ^20^ proposed quantile regression-based GWAS (QR-GWAS) summary statistics derived from the UK Biobank data. However, these works primarily address discovery rather than prediction. Recent work has demonstrated that quantile regression can be incorporated into predictive PRS modeling using individual-level genotype data, for example, through boosting-based frameworks^21^. Mefford, et al.^22^ applied conventional PRS in a quantile-prediction setting; however, the PRS weights were derived from mean-regression GWAS, and thus their model did not incorporate quantile-specific effects of SNPs. Comparatively little attention has been given to summary-statistics-based PRS approaches that leverage quantile-specific genetic effects derived from QR-GWAS for phenotype prediction.

We propose a quantile regression-based PRS (QPRS) that incorporates quantile-specific genetic effects identified from QR-GWAS into the construction of a risk score. We apply a clumping-and-thresholding (C + T) procedure to QR-GWAS summary statistics, which is the most commonly used method for computing polygenic scores. Furthermore, motivated by prior studies showing that combining information from multiple quantile estimates can improve predictive performance^23,24^, we propose using multiple QPRS scores as covariates to enhance prediction of the target phenotype. By incorporating risk scores from multiple quantiles as covariates in a phenotype prediction, QPRS captures distributional heterogeneity and efficiently pools information, thereby extending conventional PRS beyond mean-based associations. We evaluate its performance in two settings: (i) simulation studies comparing QPRS with conventional PRSs, and (ii) a real-data application using genome-wide data from the Korean Association Resource (KARE) cohort of the Korean Genome and Epidemiology Study (KoGES).

## Materials and methods

### Data description

We utilize genotype data from the KARE cohort, a population-based study within the KoGES that investigates genetic determinants of complex traits and diseases. These data are accessed via the Clinical and Omics Data Archive (CODA; https://coda.nih.go.kr) of the Korea Disease Control and Prevention Agency (KDCA). The KARE cohort is a population-based study nested within the Korean Genome and Epidemiology Study (KoGES), comprising community-dwelling adults recruited from the urban area of Ansan and the rural area of Ansung in the Republic of Korea. The baseline cohort consists of men and women, primarily middle-aged adults at enrollment. Extensive epidemiological, clinical, and lifestyle data are collected using standardized protocols, alongside genome-wide genotyping. Informed consent was obtained from all participants and/or their legal guardians. All methods were carried out in accordance with relevant guidelines and regulations. The study was approved by the Institutional Review Board of Seoul National University (IRB No. E2209/001-001).

In the real data analysis of this study, we focus on blood glucose levels and triglycerides (TG) as the primary phenotypes of interest. Blood glucose is a core metabolic trait with well-documented genetic contributions and substantial environmental modulation by factors such as diet, physical activity, and adiposity. Importantly, glucose levels exhibit pronounced distributional heterogeneity, characterized by skewness and heavy tails that reflect impaired glucose regulation and prediabetic states, even within non-diabetic populations. These characteristics make blood glucose particularly well-suited for evaluating genetic effects beyond the conditional mean, such as variance and tail-specific effects. Triglycerides similarly exhibit a highly right-skewed distribution and constitute a critical risk factor for cardiovascular disease and metabolic syndrome. TG levels also display pronounced distributional heterogeneity, making this trait equally well-suited for evaluating quantile-specific genetic effects.

The dataset initially comprises 8,840 participants and 1,573,861 SNPs. Genotypes are imputed using the 1000 Genomes Asian reference panel^25^. We apply standard quality control (QC) procedures: we exclude individuals with a genotype missingness rate $\ge$ 10% or incomplete covariate information, and filter variants with a minor allele frequency (MAF) < 1%, a Hardy–Weinberg equilibrium (HWE) *p*-value < $1 \times 10^{ - 6}$, or a missing call rate > 5%. Additionally, individuals who were already taking oral diabetes medication are excluded. After the QC procedures, 8,408 individuals and 1,573,859 SNPs are retained. The baseline demographic and clinical characteristics of the study participants from the KARE cohort are summarized in the supplementary materials. To ensure rigorous evaluation and prevent overfitting, we randomly partition these individuals into three independent subsets: a discovery set ($n = 6000$) for GWAS summary statistic derivation, a validation set ($n = 1408$) for hyperparameter tuning, model selection, and fitting of the joint prediction model, and a test set ($n = 1000$) reserved for the final assessment of predictive performance. In the real-data analysis, this partitioning process corresponds to the repetitions in fivefold cross-validation, while in the simulation study, it represents independent simulation replicates.

### Quantile-based polygenic risk score (QPRS)

A PRS is typically defined as a weighted sum of risk alleles, where the weights correspond to SNP effect sizes estimated from GWAS summary statistics^26^. PRS construction generally requires three independent datasets: a discovery set, from which summary statistics are derived; a validation set, utilized to optimize hyperparameters (e.g., *p*-value thresholds for C + T) and fit the prediction model; and an independent test set (also referred to as the target set), where the final PRS is calculated and its predictive performance is evaluated.

**Fig. 1:**
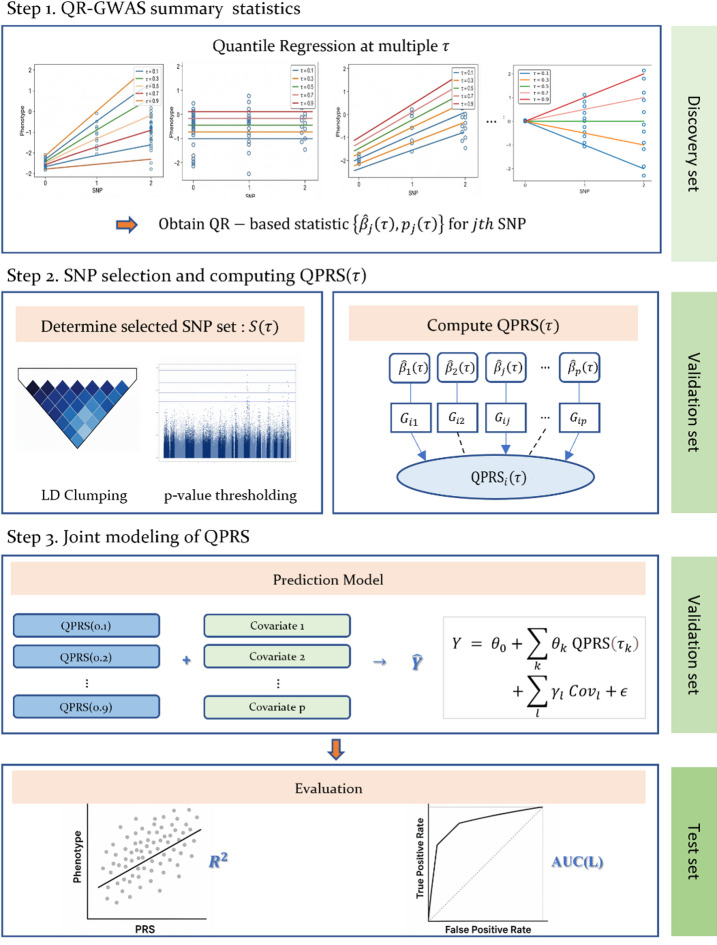
Workflow of QPRS construction. The QPRS estimates SNP effects at multiple quantiles in the discovery set, then performs C + T to select variants, computes QPRS, fits the joint prediction model in the validation set, and finally evaluates predictive performance in the held-out test set.

The proposed QPRS extends this standard framework by incorporating quantile-specific effect sizes, thereby capturing heterogeneous genetic effects across the phenotype distribution. The construction process of QPRS comprises three steps: (i) deriving summary statistics from quantile regression GWAS (QR-GWAS), (ii) calculating QPRSs using selected variants, and (iii) incorporating these scores as covariates in a downstream prediction model. Crucially, this approach leverages scores derived at multiple quantile levels to account for distributional heterogeneity and enhance predictive performance. Figure 1 provides a schematic overview of the QPRS workflow, spanning from GWAS preprocessing to quantile-specific score calculation.

#### Step 1: QR-GWAS summary statistics

In the first step, we conduct genome-wide QR on the discovery set at $\tau = 0.1, \ldots ,0.9$ to obtain GWAS summary statistics. Let $Y_{i}$ denote the phenotype, $G_{ij}$ genotype dosage for SNP $j$ (coded as 0, 1, or 2 effect allele), and $C_{i}$ covariates (e.g., age, sex, or principal components of ancestry). The $\tau$-th conditional quantile function is modeled as

$$Q_{\tau } \left( {YG_{ij} ,C_{i} } \right) = \alpha \left( \tau \right) + G_{ij} \beta_{j} \left( \tau \right) + C_{i} \gamma \left( \tau \right),$$

where $\alpha \left( \tau \right)$ is the intercept, $\beta_{j} \left( \tau \right)$ the SNP effect at quantile $\tau$, and $\gamma \left( \tau \right)$ the covariate effects at quantile $\tau$. The regression coefficients are estimated by solving the optimization problem

$$\left( {\hat{\alpha }\left( \tau \right), \hat{\beta }_{j} \left( \tau \right), \hat{\gamma }\left( \tau \right)} \right) = \mathop {argmin}\limits_{\alpha \left( \tau \right), \beta \left( \tau \right), \gamma \left( \tau \right)} \mathop \sum \limits_{i = 1}^{n} \rho_{\tau } \left( {y_{i} - \alpha \left( \tau \right) - G_{ij} \beta \left( \tau \right) - C_{i} \gamma \left( \tau \right)} \right),$$

where $\rho_{\tau } \left( u \right) = u\left( {\tau - 1_{{\{ u < 0\} }} } \right)$ is the check loss, the standard loss function in QR. Here, $1_{{\{ u < 0\} }}$ is the indicator function, which equals 1 if $u < 0$ and 0 otherwise. The loss function weights positive and negative residuals asymmetrically, allowing estimation of conditional quantiles rather than the conditional mean. Statistical significance of $\hat{\beta }_{j} \left( \tau \right)$ is evaluated using the rank-score test^27^, yielding a $p$-value $p_{j} \left( \tau \right)$ for SNP $j$. Accordingly, QR-GWAS produces summary statistics $\left\{ {\hat{\beta }_{j} \left( \tau \right), p_{j} \left( \tau \right):j = 1, \ldots ,m} \right\}$, where $m$ is the total number of SNPs.

In addition to the approach adopted here, several alternative methods are available. For instance, QR-GWAS summary statistics can be obtained from large-scale published resources^20^, when available, and $p$-values can be derived using Wald-type tests^28^, which are based on asymptotic standard errors.

#### Step 2: computing QPRS and SNP selection

We construct quantile-specific risk scores at $\tau = 0.1, \ldots , 0.9$. For a given quantile $\tau \in \left( {0,1} \right)$, the QPRS for individual $i$ is defined as

1 $${\mathrm{QPRS}}_{i} \left( \tau \right) = \mathop \sum \limits_{j \in S\left( \tau \right)} \hat{\beta }_{j} \left( \tau \right)G_{ij} ,$$

where $G_{ij}$ is the genotype dosage of SNP $j$ for individual $i$, $S\left( \tau \right)$ is the set of selected SNPs at quantile $\tau$ after the selection procedure, and $\hat{\beta }_{j} \left( \tau \right)$ is the quantile-specific effect size of the $j$-th SNP obtained from the QR-GWAS summary statistics in step 1. This formulation extends the conventional PRS by allowing genetic effects to vary across the phenotype quantiles. For notational simplicity, we suppress the individual index *i* hereafter.

To obtain $S\left( \tau \right)$, we employ a C + T strategy, a widely used and computationally straightforward method for PRS construction^29^. First, SNPs in high LD are clumped by retaining the most significant variant within each LD block. Next, variants are filtered based on a predefined *p*-value threshold. Within each LD block, we select the SNP that has the smallest *p*-value and is below the threshold. A common threshold can be imposed across all quantiles, as in this study, or specific thresholds may be applied to each quantile. In line with standard C + T procedure, we consider multiple thresholds and retain the one that yields the best predictive performance.

At each quantile, the C + T procedure is repeated to identify informative variants, yielding a quantile-specific SNP set $S\left( \tau \right)$. Because effect sizes and corresponding *p*-values differ across quantiles, the resulting SNP sets can vary accordingly. This allows QPRS to capture variants whose effects are specific to particular parts of the phenotype distribution. At the same time, variants that exert effects across the entire distribution are evaluated repeatedly through C + T procedures applied at multiple quantiles, which reduces the chance that such variants are excluded due to thresholding or LD-based pruning at any single quantile. In this sense, QPRS at multiple quantiles provides a robust aggregation of genetic signals across quantiles rather than relying on a single marginal test.

The C + T procedure can be implemented with standard tools such as PLINK, since effect-size estimates and *p*-values were already obtained in step 1. Although these estimates are derived from QR, they are provided in a format directly comparable to those from conventional linear regression–based GWAS. In this study, we apply the C + T approach to keep the procedure simple and to maintain focus on the proposed methodology. More advanced selection methods are possible and will be discussed in “Discussion” section.

#### Step 3: joint modeling of QPRS

Each QPRS($\tau$) represents the genetic contribution to a specific conditional quantile of the phenotype, thereby offering insights beyond the mean effect captured by conventional linear PRS. While individual quantile-specific scores provide valuable distributional information, their utility is further enhanced when considered together, because complex traits often exhibit heterogeneous genetic effects that vary across the outcome distribution rather than being confined to a single quantile. Integrating multiple quantile-specific scores allows the model to capture complementary aspects of genetic influence, including effects that are prominent in the tails as well as those acting more uniformly across the distribution. From a statistical perspective, aggregating information across quantiles improves robustness to misspecification of any single quantile model and increases sensitivity to heterogeneous or variance-related genetic effects, a phenomenon well documented in quantile regression literature^16,28^. We therefore adopt a joint modeling framework that simultaneously incorporates QPRS across multiple quantiles. For $\tau_{k} = k/10$, the phenotype is predicted as

$$Y = \theta_{0} + \mathop \sum \limits_{k = 1}^{9} \theta_{k} {\mathrm{QPRS}}\left( {\tau_{k} } \right) + \epsilon ,\quad \tau_{k} = 0.1, \ldots ,0.9,$$

where $\theta_{0}$ is an intercept and $\theta_{k}$ is a regression coefficient for QPRS($\tau_{k}$). This formulation integrates information across quantiles, improving predictive accuracy and robustness while preserving the interpretability of each quantile-specific effect. Unless otherwise specified, “QPRS” hereafter refers to this joint modeling framework. Additional covariates, such as sex and age, can also be incorporated into the model. The regression framework is selected based on the outcome type: linear regression for continuous traits, logistic regression for binary traits, and QR for predicting phenotype quantiles. The joint prediction model is fitted on the validation set, and its performance is evaluated on the held-out test set.

The key distinction between QPRS and conventional linear PRS is that multiple genetic scores can be incorporated as covariates in the predictive model. Unlike the conventional approach representing genetic effects only on single mean-based score, QPRS employs multiple quantile-based scores.

Specifically, this joint modeling strategy aligns with the principles of composite quantile regression^24^ and ensemble learning. By aggregating information across multiple quantiles, the model effectively ‘borrows strength’ from stable central quantiles to regularize the estimation of tail effects, which often suffer from high variability due to data sparsity. Furthermore, the set of quantile-specific scores acts as a basis expansion, allowing the model to approximate the complex genetic architecture more comprehensively than any single-quantile estimator.

Thus, QPRS captures heterogeneity in genetic effects, enabling effective application to various prediction tasks, such as predicting conditional quantiles of the phenotype or its mean. Each QPRS($\tau$) reflects genetic effects on a different quantile of the phenotype, and together they enhance prediction accuracy. As shown in “Results” section, this joint modeling improves predictive accuracy.

### Simulation study

We evaluate the performance of the proposed QPRS method using Monte Carlo simulations. We also include a conventional linear PRS derived from mean regression using the C + T procedure, hereafter referred to as the linear regression-based PRS (LPRS). To emulate realistic genetic architecture, we generate semi-synthetic datasets by sampling genotypes from the KARE dataset. A detailed description of the KARE dataset is provided in “Data description” section. For the simulation study, we reduce the dimensionality of the genotype data via additional LD pruning. This step mitigates spurious correlations due to LD and preserves causal SNPs, enabling a more reliable assessment of whether the QR-based C + T procedure can accurately identify causal variants. Applying a sliding window of 50 SNPs, a step size of 5 SNPs, and an LD threshold of $r^{2} < 0.2$, we retain 154,455 SNPs.

For evaluation purposes, we partition the dataset into discovery, validation, and test sets. Parameter tuning, specifically threshold selection for the C + T procedure, is performed using the validation set. The selected model is then applied to the test set to compute evaluation metrics, ensuring an unbiased estimation of performance. We simulate phenotypes under two distinct schemes; the detailed simulation design and evaluation metrics are outlined below.

GWAS summary statistics are derived separately for each method. For LPRS, we perform GWAS using linear mean regression in the discovery set. For QPRS, we conduct QR-GWAS across multiple quantile levels. In both approaches, SNPs are selected via the C + T procedure. LD clumping is implemented with a 1 Mb sliding window: within each window, the SNP with the lowest *p*-value is retained as the index SNP, and all variants with a pairwise $r^{2} > 0.5$ relative to the index are removed. This configuration is standard in PRS analyses and corresponds to the default settings in PLINK^30^.

To account for the sensitivity of C + T to the choice of *p*-value thresholds, we consider a predefined grid of candidate thresholds. We select different grids of *p*-value thresholds for QPRS and LPRS to account for the distinct distributions of association statistics generated by each method. Note that *p*-values from a standard linear regression-based GWAS (used for LPRS) are not directly comparable to those from the QR-GWAS, which assesses significance at specific quantiles. To ensure a fair comparison, we establish separate but functionally equivalent grids for each method. For LPRS, the grid is: $\left\{ {1 \times 10^{ - 6} , 1 \times 10^{ - 5} , 5 \times 10^{ - 5} } \right.$, $1 \times 10^{ - 4} , 5 \times 10^{ - 4} , 1 \times 10^{ - 3}$, $\left. {5 \times 10^{ - 3} , 1 \times 10^{ - 2} } \right\}$, while for QPRS, a wider range starting from a more stringent cutoff is used: $\left\{ {1 \times 10^{ - 8} , 5 \times 10^{ - 8} ,1 \times 10^{ - 7} } \right.$, $1 \times 10^{ - 6} , 1 \times 10^{ - 5} ,1 \times 10^{ - 4}$, $\left. { 1 \times 10^{ - 3} , 1 \times 10^{ - 2} } \right\}.$ Each grid is designed to span an appropriate spectrum for its respective method, from highly stringent cutoffs yielding few or no SNPs to ones yielding a substantially large number (more than 500 SNPs included). For QPRS, the best-performing threshold is applied uniformly across all quantiles to maintain consistency.

#### Simulation scheme 1: with vQTL

The first simulation setting considers the presence of variance quantitative trait loci (vQTLs), genetic loci that influence the variability of a phenotype rather than its mean. A vQTL effect, by increasing or decreasing phenotypic variance, naturally manifests as heterogeneous genetic effects across the distribution; for instance, a variance-increasing allele will have a larger effect on the upper and lower tails than at the median. This setting allows us to directly assess the ability of QPRS to detect and leverage these heterogeneous effects for improved prediction.

Our simulation design is adapted from Choi and O’Reilly^31^, with modifications to incorporate heterogeneity. Let $X$ denote the genotype matrix. We generate simulated phenotypes as

$$Y_{raw} = X\beta + \left( {X\gamma } \right)\epsilon ,$$

where $\beta$ denotes the mean effect and $\gamma$ the variance effect. Note that when the error term $\epsilon$ has mean zero, $\gamma$ does not affect the mean of the phenotype but influences its variance, thereby inducing quantile-specific genetic effects. Specifically, the quantile effect sizes of SNPs are given by

$$\beta \left( \tau \right) = \beta + \gamma Q_{\epsilon } \left( \tau \right),$$

where $Q_{\epsilon } \left( \tau \right)$ is the $\tau$-th quantile of the error distribution. For $\beta$, $p_{1}$ (the number of causal SNPs with mean effects) causal SNPs are each assigned an effect size of $5/p_{1}$, with all other SNPs set to zero. For $\gamma$, 5 causal SNPs are each assigned an effect size of 2, with the remaining SNPs set to zero. There are two practical reasons for this configuration. First, given that genotype dosages are discrete (0, 1, 2) and many variants exhibit limited counts of minor-allele homozygotes in finite samples, assigning very small $\gamma$ values would yield weak modulation of the variance term, resulting in insufficient heterogeneity to evaluate quantile-specific effects. Second, if variance effects are dispersed across a large number of variants, their collective variability tends to be attenuated, thereby reducing the heterogeneous contrast that drives quantile-dependent signals. Therefore, we fix the effect size of vQTLs and vary $p_{1}$ across 5, 10, 50, and 100. By holding the total effect size constant and varying $p_{1}$, we contrast sparse architectures, characterized by a few large-effect variants, with polygenic architectures, characterized by many small-effect variants. For the error term $\epsilon$, we consider three distributions: standard normal, Student’s *t* with 5 degrees of freedom, and standard log-normal.

To adjust for population structure, we regress the simulated phenotype $Y_{raw}$ on the first five principal components ($PC_{1}$,…$,PC_{5}$) of the genotype data:

2 $$Y_{raw} = \alpha + \eta PC_{{\left\{ {1:5} \right\}}} + \delta ,$$

where $\alpha$ is an intercept, and $\eta$ a coefficient vector of principal components, and ${ delta }$ an error term. Residuals from model (2) are used as the final phenotype $Y$. This adjustment follows the approach of Choi and O’Reilly^31^.

#### Scheme 2: with outliers

The second simulation setting considers phenotypes contaminated with massive outliers. Because linear regression is highly sensitive to outliers, it may fail in this scenario, whereas QR provides a more robust alternative and is expected to achieve higher predictive accuracy.

We generate phenotypes from the linear model

$$Y_{raw} = X\beta + \epsilon$$

where now $\beta \left( \tau \right) = \beta$ for all $\tau \in \left( {0,1} \right)$, indicating that the genetic effect is homogeneous across the phenotype distribution. We assign effect sizes to 50 randomly selected causal SNPs by sampling from the standard normal distribution $N\left( {0,1} \right)$, with all other SNP effects set to zero.

To introduce contamination, we multiply phenotype values for 1%, 2%, 5%, or 10% of the observations by factors of 5, 10, 20, or 50. As in Eq. (2), we use residuals from principal component regression on the first five PCs as the final phenotype.

#### Evaluation metric

We evaluate the performance of the proposed QPRS relative to LPRS in terms of variant selection capability and predictive accuracy. To assess the efficacy of SNP selection, we employ precision, recall, and the size of the selected SNP set (denoted as $\left| S \right|$). Precision measures the reliability of detected signals, defined as the proportion of true causal variants among the selected SNPs, while recall quantifies sensitivity, representing the proportion of total causal variants correctly identified. Additionally, $\left| S \right|$ provides insight into model sparsity by indicating the total number of variants retained following the C + T procedure.

Next, we evaluate the predictive accuracy of the risk scores. The goal is to determine how effectively the scores predict phenotype outcomes. In scheme 1, we evaluate the ability of QPRS and LPRS to predict phenotype quantiles at τ = 0.1, 0.5, and 0.9. We hypothesize that QPRS will exhibit superior performance when targeting the lower ($\tau = 0.1$) or upper ($\tau = 0.9$) quantiles, where vQTL effects are expected to be present, but not when targeting the median, which is primarily influenced by mean effects. After fitting QR models that include QPRS (or LPRS) along with covariates, predictive accuracy is measured by the mean squared error (MSE) between the estimated and true quantiles. Here, the true quantile function is defined as

$$Q_{Y} \left( {\tau {|}X} \right) = X\beta + \left( {X\gamma } \right)Q_{\epsilon } \left( \tau \right)$$.

In scheme 2, we assess QPRS and LPRS by their accuracy in predicting the mean and median of the phenotype. After fitting linear regression with QPRS (or LPRS) and covariates, accuracy is quantified by the MSE between the true mean function, $X\beta$, and the fitted values. Each simulation is repeated 25 times, and the reported results are the averages of the evaluation metrics.

### Real data analysis

#### Analysis procedure

To assess the generalizability of the QPRS framework, we analyze two metabolic traits characterized by right-skewed distributions: 2-h post-OGTT blood glucose level and TG. We implement fivefold cross-validation, stratified by glucose status (> 200 mg/dL vs. ≤ 200 mg/dL). In each iteration, the joint prediction model is fitted on the validation set, and predictive performance is evaluated on the held-out test fold. The final reported performance represents the average across the five independent test folds. Covariates included in the analysis are sex, age, BMI, smoking status, and the first 10 principal components ($PC_{1}$,…$,PC_{10}$).

QPRS is computed following the same procedure as in simulation design. LD clumping is applied in 1 Mb windows, excluding SNPs with $r^{2} > 0.5$ relative to the index SNP. Six $p$-value thresholds for C + T procedure ($5 \times 10^{ - 2} , 1 \times 10^{ - 2} , 5 \times 10^{ - 3} , 1 \times 10^{ - 3} , 1 \times 10^{ - 4} ,1 \times 10^{ - 5}$) are considered. More stringent thresholds are avoided as they yielded almost no variants. The optimal threshold in each fold is selected based on predictive performance, measured by $R^{2}$ for the continuous trait and AUC for the binary trait.

#### Evaluation

We evaluate performance for two continuous traits, 2-h post-OGTT blood glucose and TG, and a binary trait defined by dichotomizing blood glucose levels at 200 mg/dL. Clinically, blood glucose levels serve as a cornerstone for diagnosing type 2 diabetes and prediabetes, with a threshold of 200 mg/dL indicating high-risk status^32^. Similarly, TG levels are a critical risk factor for cardiovascular diseases and a key component of metabolic syndrome. The distributions of both traits are typically right-skewed, characterized by a tail of high-risk individuals^33^. This distributional heterogeneity suggests that genetic determinants may exert stronger or distinct effects in the high-risk tail compared to the population mean. Consequently, by modeling quantile-specific effects, we anticipate that QPRS offers advantages over conventional mean-based methods, particularly for right-skewed traits such as TG where distributional heterogeneity in genetic effects is most pronounced.

For each continuous trait, we fit two linear regression models: one including only the PRS as a predictor, and another including the PRS along with covariates (sex, age, BMI, smoking status, and the first 10 principal components, $PC_{1}$,…$,PC_{10}$). Model performance is assessed using the coefficients of determination, $R^{2}$. For the binary trait, we fit logistic regression with the same two configurations (PRS only, and PRS with covariates). In all cases, lasso-penalized regression, linear for continuous traits and logistic for the binary trait, is employed to estimate the prediction model coefficients, thereby providing regularization that stabilizes out-of-sample performance across all methods under comparison. Model performance is evaluated using the area under the curve (AUC). We report AUC on two distinct scales to provide a comprehensive assessment. The first is the standard observed-scale AUC, which measures the model’s ability to discriminate between cases and controls within our specific sample. The second is the liability-scale AUC (AUCL)^34^, which is calculated based on the underlying continuous liability threshold model for complex traits. The purpose of using AUCL is to adjust the performance metric for the population prevalence of the disease, thereby mitigating potential biases that can arise from study designs, particularly case–control ascertainment.

#### Comparison methods

We compare QPRS with five benchmark PRS methods: LPRS, lassosum^4^, LDpred^5^, SBLUP^6^, and PRS-CS^7^. All reported metrics are averaged across the five cross-validation folds. We conduct QR analyses using the *quantreg* package^35^ in R, and fit lassosum models with the *lassosum* package^4^ in R version 4.4.0 (R Foundation for Statistical Computing, Vienna, Austria). We perform standard linear regression-based GWAS using PLINK (version 1.90b7.2). For LDpred and SBLUP, we use LDpred^5^ (version 1.0.10) and GCTA^36^, respectively, both of which use GWAS summary statistics generated by BOLT-LMM^37^ as input. For PRS-CS, we employ the Python-based implementation available at https://github.com/getian107/PRScs, using the 1000 Genomes Project Phase 3 reference panel.

## Results

### Simulation results

#### Scheme 1: with vQTL

Table 1 summarizes the SNP selection performance of LPRS, QPRS, and the single-quantile model QPRS(0.5) under simulation scheme 1. In sparse settings ($p_{1} = 5$ and 10), QPRS consistently achieves substantially higher recall than LPRS across most error distributions and genetic architectures, effectively recovering the majority of causal variants. In contrast, LPRS exhibits markedly lower recall, indicating limited sensitivity to quantile-specific genetic effects. This improvement in recall is accompanied by a larger size of the selected SNP set, reflecting the aggregation of signals across multiple quantiles. QPRS yields lower precision than LPRS in certain scenarios, illustrating the inherent precision–recall tradeoff. As the number of causal mean-effect variants increases (*p*₁ = 50 and 100), all methods exhibit a decline in both precision and recall, consistent with the well-known limitations of C + T-based selection in highly polygenic architectures. Nevertheless, QPRS maintains higher recall than LPRS across these settings, suggesting that quantile-specific information facilitates signal recovery even when individual effects are weak.

**Table 1:** Precision and recall of SNP selection for LPRS, QPRS, and single-quantile QPRS(0.5) in scheme 1 across varying numbers of causal SNPs $(p_{1} )$ and error distributions.

$p_{1}$	Score	$\epsilon \sim N\left( {0,1} \right)$			$\epsilon \sim t\left( 5 \right)$			$\epsilon \sim logN\left( {0,1} \right)$		
		Precision	Recall	$\left| S \right|$	Precision	Recall	$\left| S \right|$	Precision	Recall	$\left| S \right|$
5	LPRS	0.532 (0.056)	0.420 (0.016)	11.28 (1.57)	0.656 (0.055)	0.340 (0.024)	6.60 (0.85)	0.216 (0.050)	0.204 (0.026)	44.72 (13.00)
	QPRS	0.219 (0.023)	0.848 (0.024)	56.00 (8.18)	0.171 (0.024)	0.820 (0.026)	112.20 (26.88)	0.102 (0.016)	0.896 (0.017)	244.04 (78.54)
	QPRS(0.5)	0.604 (0.051)	0.392 (0.019)	8.20 (0.96)	0.631 (0.047)	0.388 (0.020)	7.56 (0.93)	0.462 (0.038)	0.780 (0.023)	21.32 (2.69)
10	LPRS	0.667 (0.075)	0.237 (0.026)	20.60 (8.59)	0.387 (0.085)	0.165 (0.021)	139.64 (50.96)	0.007 (0.003)	0.051 (0.017)	210.48 (61.08)
	QPRS	0.104 (0.019)	0.616 (0.035)	248.44 (68.06)	0.103 (0.020)	0.549 (0.034)	372.28 (131.21)	0.045 (0.008)	0.595 (0.037)	471.80 (126.16)
	QPRS(0.5)	0.817 (0.048)	0.280 (0.032)	7.00 (1.71)	0.671 (0.084)	0.200 (0.033)	13.08 (3.64)	0.387 (0.036)	0.419 (0.030)	25.32 (7.17)
50	LPRS	0.002 (0.001)	0.004 (0.002)	98.28 (32.28)	0.001 (0.001)	0.007 (0.003)	208.48 (60.84)	0.001 (0.000)	0.004 (0.002)	291.52 (68.09)
	QPRS	0.063 (0.013)	0.095 (0.006)	716.52 (196.54)	0.062 (0.018)	0.092 (0.004)	738.84 (177.92)	0.025 (0.005)	0.100 (0.005)	1000.16 (284.83)
	QPRS(0.5)	0.018 (0.015)	0.004 (0.003)	18.00 (3.20)	0.000 (0.000)	0.000 (0.000)	11.52 (1.783)	0.326 (0.042)	0.079 (0.003)	26.80 (6.54)
100	LPRS	0.003 (0.002)	0.005 (0.002)	253.32 (66.92)	0.004 (0.003)	0.003 (0.001)	167.48 (47.89)	0.003 (0.001)	0.003 (0.001)	152.44 (50.47)
	QPRS	0.077 (0.019)	0.045 (0.002)	499.40 (158.41)	0.019 (0.006)	0.053 (0.003)	1160.44 (223.09)	0.023 (0.006)	0.059 (0.006)	1524.88 (510.80)
	QPRS(0.5)	0.014 (0.014)	0.000 (0.000)	7.72 (1.43)	0.008 (0.008)	0.001 (0.001)	6.40 (1.46)	0.155 (0.025)	0.043 (0.002)	129.68 (63.98)

Standard errors from 25 iterations are reported in parentheses.

The comparison with the single-quantile model, QPRS(0.5), further shows the benefits of utilizing multi-quantile risk scores. While QPRS(0.5) tends to attain relatively higher precision in sparse genetic architectures ($p_{1} = 5,10$), its recall remains substantially lower than that of the full QPRS, particularly under heavy-tailed or skewed error distributions. This pattern indicates that single-quantile approaches preferentially capture central effects but often miss distribution-specific signals manifesting away from the median. Although a single-quantile approach may suffice for identifying specifically associated variants, we advocate for our proposed joint framework, integrating scores across multiple quantiles, especially when the primary objective is to maximize predictive performance by capturing a broader spectrum of genetic signals.

Table 2 presents the predictive performance of LPRS, QPRS, and the single-quantile QPRS($\tau$) under scheme 1. These results demonstrate the distinct advantages of the joint use of QPRS, particularly in capturing tail-specific genetic effects driven by vQTLs. First, in predicting tail quantiles ($\tau = 0.1, 0.9$), joint QPRS consistently yields the lowest MSE across diverse genetic architectures and error distributions. Notably, the joint model outperforms not only the LPRS but also the corresponding single-quantile model QPRS($\tau$). This finding suggests that integrating information across multiple quantiles provides more stable and accurate predictions than relying solely on the target quantile. By borrowing strength from the entire distribution, the joint model effectively reduces estimation variance and enhances the identification of heterogeneous genetic signals. This performance gap is particularly pronounced under non-normal error distributions. In the presence of heavy-tailed or skewed errors, where LPRS often fails to capture the underlying data structure, QPRS demonstrates substantial robustness and superior predictive accuracy.

**Table 2:** Mean of MSE of LPRS, QPRS, and single-quantile QPRS($$\tau$$) in simulation scheme 1 across varying numbers of causal SNPs ($$p_{1} )$$ and error distributions.

$p_{1}$	$\tau$	$\epsilon \sim N\left( {0,1} \right)$			$\epsilon \sim t\left( 5 \right)$			$\epsilon \sim logN\left( {0,1} \right)$		
		LPRS	QPRS	QPRS($\tau$)	LPRS	QPRS	QPRS($\tau$)	LPRS	QPRS	QPRS($\tau$)
5	0.1	23.78 (1.33)	18.34 (1.64)	23.81 (1.44)	26.42 (1.37)	20.75 (1.79)	27.32 (1.51)	35.30 (1.73)	25.84 (1.78)	33.16 (2.12)
	0.5	8.38 (0.88)	8.62 (0.87)	8.51 (0.91)	8.18 (0.83)	8.19 (0.88)	8.33 (0.86)	8.38 (0.67)	9.31 (0.84)	8.90 (0.77)
	0.9	15.24 (0.94)	9.99 (1.09)	14.69 (0.90)	18.37 (0.95)	12.34 (1.33)	16.93 (1.04)	35.93 (2.17)	28.84 (2.87)	34.86 (2.22)
10	0.1	23.07 (1.05)	18.10 (1.20)	22.74 (1.03)	28.07 (1.22)	21.81 (1.60)	27.20 (1.17)	34.85 (1.35)	24.79 (1.59)	33.30 (1.35)
	0.5	7.66 (0.63)	7.38 (0.60)	7.63 (0.62)	7.85 (0.67)	7.87 (0.71)	8.03 (0.69)	7.52 (0.53)	8.80 (0.54)	7.81 (0.56)
	0.9	14.59 (0.63)	11.27 (1.14)	13.57 (0.64)	18.48 (0.80)	12.50 (1.40)	16.92 (1.01)	37.24 (2.04)	30.49 (2.61)	35.85 (2.01)
50	0.1	23.71 (0.87)	22.34 (0.79)	22.28 (0.87)	28.13 (1.03)	27.56 (1.50)	26.96 (1.35)	33.83 (0.73)	24.48 (1.56)	32.00 (1.08)
	0.5	7.82 (0.23)	7.96 (0.26)	7.93 (0.29)	7.82 (0.30)	8.57 (0.30)	8.16 (0.23)	7.25 (0.25)	8.44 (0.29)	7.65 (0.26)
	0.9	14.80 (0.51)	15.00 (0.85)	13.62 (0.56)	18.55 (0.89)	18.81 (1.18)	17.54 (1.01)	35.96 (1.71)	28.59 (2.39)	33.05 (1.91)
100	0.1	21.56 (0.70)	19.98 (0.82)	20.96 (0.72)	26.16 (0.92)	22.61 (0.92)	25.79 (1.05)	31.99 (0.73)	24.74 (1.40)	29.73 (0.73)
	0.5	7.20 (0.22)	7.80 (0.24)	7.61 (0.25)	7.52 (0.18)	7.72 (0.18)	7.45 (0.19)	6.48 (0.22)	7.16 (0.23)	6.76 (0.27)
	0.9	13.78 (0.50)	13.02 (1.01)	12.85 (0.63)	16.71 (0.85)	14.32 (1.07)	15.77 (0.89)	33.98 (1.56)	29.34 (2.74)	31.83 (1.65)

QPRS($\tau$) denotes the risk score derived solely from the specific target quantile. Standard errors for 25 iterations are reported in parentheses.

At the median, where the theoretical impact of vQTLs is negligible, the performance of QPRS remains comparable to that of LPRS. Although LPRS is theoretically optimal for the conditional mean in the absence of variance effects, QPRS exhibits no significant loss of efficiency. This indicates that the proposed method is robust, maintaining competitive performance for central tendencies while providing substantial gains in the tails, where genetic heterogeneity is most pronounced.

Overall, the combined evidence from Tables 1 and 2 demonstrates that QPRS enhances SNP recovery under heterogeneous genetic architectures and that this improvement directly translates into better quantile-specific prediction, particularly for extreme phenotypic outcomes where mean-based PRS are inherently limited.

#### Scheme 2: with outliers

Table 3 summarizes the predictive performance, and Table 4 presents variant selection metrics under varying degrees of outlier contamination. The results illustrate a distinct trade-off between predictive efficiency and robustness across the compared methods. In Table 3, under mild-to-moderate contamination scenarios (1–5% outliers with shift magnitudes of 5 or 10), the joint QPRS model consistently achieves the lowest MSE, outperforming both the LPRS and the median-only model, QPRS(0.5). For instance, with 1% outliers of magnitude 5, joint QPRS yields an MSE of 19.20, notably lower than the 23.21 observed for LPRS and 23.35 for QPRS(0.5). This performance advantage suggests that when the influence of outliers is moderate, the joint modeling approach successfully borrows strength across quantiles to refine effect estimates, thereby maximizing predictive accuracy. However, in settings characterized by extreme contamination (e.g., 10% outliers with magnitude 50), the stability of median regression becomes paramount. In such extreme scenarios, the MSE of joint QPRS inflates due to instability in tail quantile estimates. However, under extreme contamination scenarios, this advantage reverses; whereas QPRS(0.5) maintains robust performance, establishing the median-only model as a safer alternative when the phenotype distribution is severely distorted.

**Table 3:** Mean of MSEs for mean regression and median regression in scheme 2 across varying outlier proportions and magnitudes.

Outlier (%)	Outlier degree	5		10		20		50	
	Method	Mean	Median	Mean	Median	Mean	Median	Mean	Median
1	LPRS	23.21 (1.77)	24.35 (1.93)	23.10 (1.76)	25.10 (2.11)	23.08 (1.76)	26.80 (2.47)	27.08 (1.80)	36.37 (4.05)
	QPRS	19.20 (1.83)	20.13 (1.99)	19.71 (1.84)	21.35 (2.12)	21.11 (1.85)	24.03 (2.61)	30.33 (2.15)	32.80 (4.07)
	QPRS(0.5)	23.35 (1.81)	24.24 (1.93)	23.13 (1.80)	25.20 (2.14)	23.48 (1.81)	27.65 (2.56)	26.84 (1.92)	36.64 (4.02)
2	LPRS	23.20 (1.75)	24.67 (2.03)	23.17 (1.79)	26.76 (2.43)	24.35 (1.98)	31.24 (3.31)	33.60 (2.92)	52.09 (7.08)
	QPRS	19.24 (1.83)	20.57 (2.06)	20.92 (1.93)	23.50 (2.52)	24.77 (2.05)	28.76 (3.51)	51.47 (3.75)	49.19 (7.06)
	QPRS(0.5)	23.34 (1.82)	24.85 (2.09)	23.59 (1.86)	26.93 (2.47)	25.07 (1.97)	32.29 (3.30)	33.80 (3.03)	52.47 (6.99)
5	LPRS	23.60 (1.86)	27.39 (2.53)	24.77 (1.99)	33.83 (3.68)	29.63 (2.61)	50.25 (6.58)	66.61 (6.74)	130.82 (21.05)
	QPRS	20.13 (1.88)	23.42 (2.56)	23.57 (2.11)	30.35 (3.84)	33.91 (2.69)	47.17 (6.71)	115.73 (9.13)	128.88 (21.19)
	QPRS(0.5)	23.76 (1.82)	27.55 (2.49)	24.66 (2.03)	33.89 (3.65)	29.30 (2.64)	51.43 (6.71)	57.72 (6.48)	132.39 (21.06)
10	LPRS	23.84 (1.87)	32.71 (3.49)	26.09 (2.02)	48.94 (6.33)	37.66 (3.32)	98.40 (15.08)	129.00 (18.86)	370.57 (63.46)
	QPRS	21.12 (1.88)	28.42 (3.52)	29.03 (2.04)	46.49 (6.59)	57.45 (4.53)	94.80 (15.29)	259.99 (18.03)	367.39 (63.57)
	QPRS(0.5)	23.94 (1.88)	32.77 (3.48)	26.21 (2.04)	50.14 (6.50)	35.36 (3.58)	98.55 (15.12)	90.03 (10.42)	371.39 (63.60)

Standard errors from 25 iterations are provided in parentheses.

**Table 4:** Precision and recall in scheme 2 across varying outlier proportions and magnitudes.

Outlier (%)	Outlier degree	5			10			20			50		
	Method	Precision	Recall	$\left| S \right|$	Precision	Recall	$\left| S \right|$	Precision	Recall	$\left| S \right|$	Precision	Recall	$\left| S \right|$
1	LPRS	0.291 (0.025)	0.522 (0.019)	142.20 (40.52)	0.343 (0.020)	0.414 (0.017)	72.08 (10.48)	0.365 (0.030)	0.231 (0.015)	44.84 (9.98)	0.169 (0.036)	0.080 (0.010)	93.84 (40.86)
	QPRS	0.378 (0.027)	0.444 (0.017)	73.56 (11.08)	0.383 (0.027)	0.432 (0.015)	76.48 (15.05)	0.370 (0.030)	0.441 (0.020)	88.28 (17.61)	0.335 (0.034)	0.460 (0.020)	110.80 (21.25)
	QPRS(0.5)	0.491 (0.022)	0.364 (0.014)	49.60 (5.03)	0.495 (0.021)	0.368 (0.015)	69.72 (11.86)	0.484 (0.024)	0.369 (0.020)	66.04 (14.08)	0.469 (0.026)	0.389 (0.018)	62.52 (10.18)
2	LPRS	0.312 (0.025)	0.484 (0.018)	121.40 (32.13)	0.368 (0.029)	0.353 (0.018)	65.64 (11.28)	0.442 (0.051)	0.179 (0.017)	69.20 (35.26)	0.199 (0.052)	0.081 (0.012)	187.24 (64.61)
	QPRS	0.404 (0.024)	0.418 (0.150)	63.12 (10.88)	0.365 (0.028)	0.436 (0.016)	84.28 (16.57)	0.355 (0.028)	0.435 (0.014)	91.36 (18.38)	0.326 (0.035)	0.467 (0.019)	254.12 (97.60)
	QPRS(0.5)	0.512 (0.019)	0.350 (0.015)	56.56 (9.05)	0.489 (0.024)	0.366 (0.015)	58.48 (9.88)	0.491 (0.024)	0.365 (0.014)	61.72 (12.53)	0.449 (0.031)	0.390 (0.016)	69.20 (9.02)
5	LPRS	0.351 (0.030)	0.451 (0.016)	91.40 (15.80)	0.418 (0.039)	0.288 (0.018)	58.12 (13.21)	0.472 (0.058)	0.157 (0.015)	66.80 (35.78)	0.371 (0.047)	0.086 (0.014)	153.32 (64.00)
	QPRS	0.388 (0.020)	0.418 (0.015)	60.00 (6.13)	0.371 (0.024)	0.416 (0.011)	66.24 (7.13)	0.363 (0.026)	0.426 (0.015)	75.52 (12.74)	0.313 (0.033)	0.455 (0.018)	229.72 (97.91)
	QPRS(0.5)	0.507 (0.020)	0.348 (0.016)	63.76 (10.89)	0.509 (0.019)	0.346 (0.010)	55.76 (8.64)	0.510 (0.019)	0.361 (0.016)	75.76 (12.72)	0.478 (0.031)	0.388 (0.016)	77.80 (15.86)
10	LPRS	0.335 (0.030)	0.418 (0.018)	87.52 (13.28)	0.391 (0.044)	0.293 (0.021)	114.76 (46.66)	0.492 (0.054)	0.182 (0.017)	35.76 (8.80)	0.501 (0.056)	0.103 (0.012)	25.80 (9.47)
	QPRS	0.341 (0.022)	0.418 (0.016)	69.84 (6.51)	0.312 (0.025)	0.410 (0.017)	86.72 (13.16)	0.234 (0.027)	0.402 (0.016)	139.68 (22.91)	0.127 (0.026)	0.451 (0.020)	591.52 (155.47)
	QPRS(0.5)	0.508 (0.019)	0.353 (0.014)	49.36 (6.10)	0.514 (0.022)	0.338 (0.016)	46.80 (6.10)	0.524 (0.019)	0.335 (0.016)	41.80 (3.17)	0.482 (0.025)	0.370 (0.018)	42.76 (3.31)

Standard errors from 25 iterations are provided in parentheses. $\left| S \right|$ denotes the size of the selected SNP set.

The analysis of precision and recall further elucidates the mechanisms driving these predictive patterns. As shown in Table 4, LPRS demonstrates high sensitivity to outlier magnitude; while its precision remains moderate, its recall declines substantially as the outlier magnitude increases, dropping to approximately 0.08 in the most extreme settings. This confirms that ordinary least squares-based association tests lose significant statistical power in the presence of heavy tails, resulting in a reduced capacity to identify causal variants. In contrast, the joint QPRS maintains a stable and relatively high recall across all scenarios, reflecting its capacity to preserve signal detection under heterogeneity. However, its precision degrades sharply under severe contamination (dropping to 0.127 at 10% outliers, degree 50), indicating that noise accumulation from tail quantiles leads to an increased rate of false positives. Meanwhile, QPRS(0.5) employs a conservative selection strategy, consistently yielding the highest precision but moderate recall across settings.

A key methodological insight from our simulation study concerns the trade-off between predictive efficiency and robustness. While the joint QPRS is superior under mild-to-moderate contamination by borrowing strength across quantiles, it is susceptible to bias under extreme outlier scenarios. Theoretically, this vulnerability stems from the lower breakdown point of tail quantiles compared to the median. When the data contain gross contamination, the biased estimates from the tails propagate through the joint summation, destabilizing the final prediction. Therefore, we recommend joint QPRS as the default strategy for maximizing predictive power in traits with typical skewness or mild outliers. However, for rare cases where diagnostic metrics (e.g., Rosner’s test^38^ or high kurtosis) indicate extreme, pathological contamination, we suggest falling back to the median-only approach to ensure maximum stability.

Notably, the observed performance gap is driven by the robustness of the variant selection mechanism, rather than by the total number of variants available for analysis. Although the input dimensionality was identical for both methods, LPRS selects a substantially larger number of variants with low precision in the presence of outliers, indicating an inability to distinguish causal signals from noise. In contrast, QPRS effectively filters out these artifactual associations, ensuring that the risk score is constructed primarily from true signals. This confirms that the superior predictive accuracy of QPRS stems from its qualitative advantage in identifying valid genetic markers under contamination.

Under outlier contamination, threshold-based methods like C + T are strictly dependent on the validity of GWAS summary statistics. While OLS-derived estimates crumble under assumption violations, leading to false negatives during thresholding, QPRS provides the robust inputs necessary for the C + T procedure to function correctly.

Overall, the comparison of schemes 1 and 2 illustrates that QPRS is particularly advantageous when genetic effects vary across quantiles, as in variance QTL (vQTL) settings. In contrast, under homogeneous mean-effect models subject to outlier contamination, its main strength lies in robustness to contamination and in preserving specificity, rather than in consistently achieving higher sensitivity.

### Real data analysis results

The phenotypes analyzed in this study are 2-h post-OGTT blood glucose levels and triglyceride (TG). The distributions of both phenotypes are right-skewed. This skewness, together with evidence of heterogeneous variance across the phenotype distribution, suggests potential limitations of mean-based linear regression for fully characterizing genetic effects on these traits. Moreover, despite applying a logarithmic transformation, Kolmogorov–Smirnov tests indicate that the distributions still deviate significantly from normality. Our proposed methods leverage QR to capture distributional heterogeneity and tail-specific genetic effects in the observed data, offering robustness to outliers and departures from homoscedasticity, rather than relying solely on assumptions about the conditional mean.

**Fig. 2:**
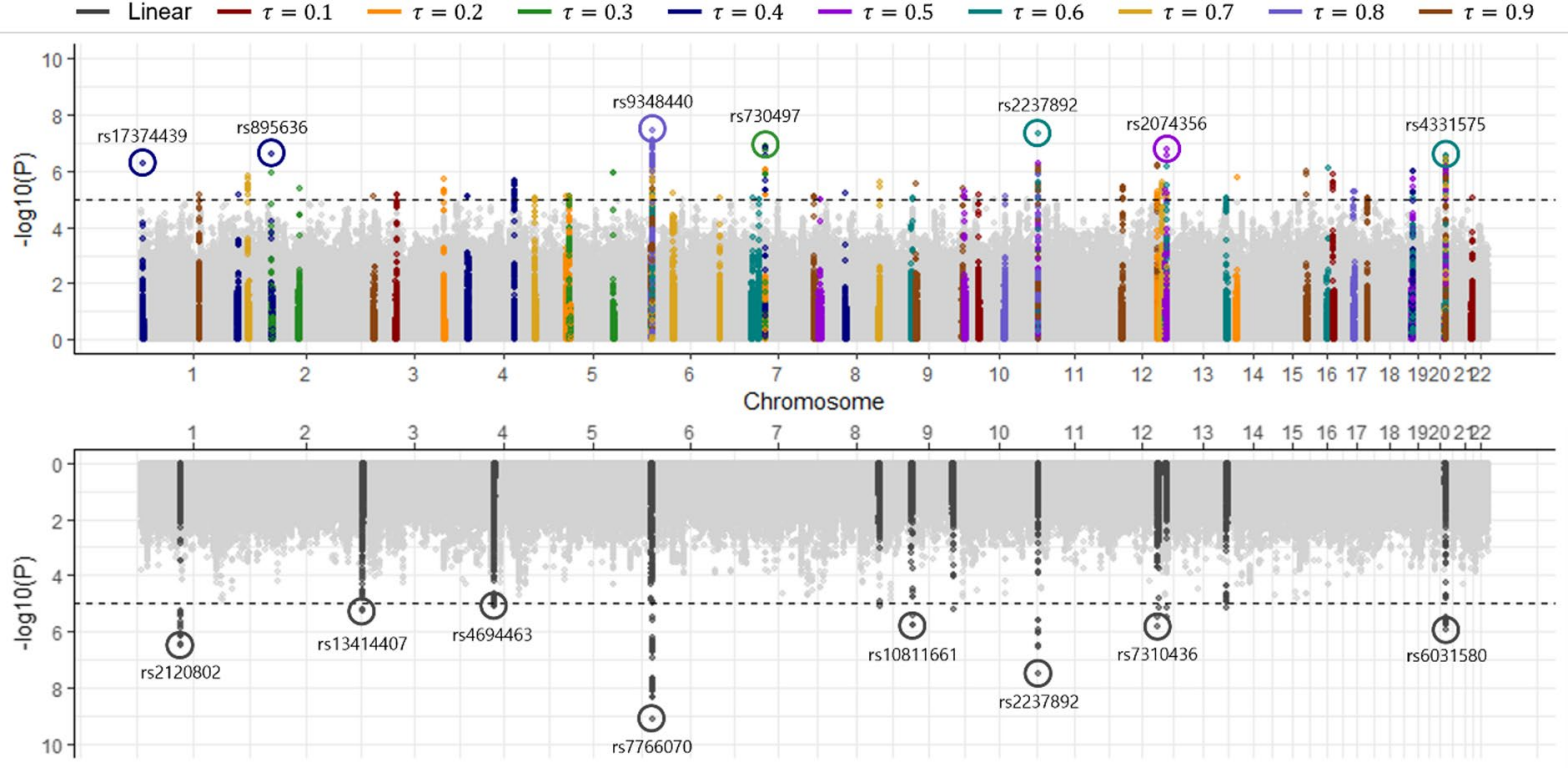
Manhattan plots of GWAS for 2-h post-OGTT glucose levels. The top panel displays the overlaid $- \log_{10} p$ from QR-GWAS across nine quantiles ($\tau = 0.1, \ldots ,0.9$). The circled markers represent representative lead SNPs identified by the QR-GWAS, with the color of each circle corresponding to the specific quantile where the variant exhibited the most significant association. The bottom panel shows association results from the standard linear regression-based GWAS, where the circled markers indicate the top loci identified by the mean-based model. The horizontal dashed line indicates the significance threshold ($p = 1 \times 10^{ - 5}$).

To illustrate the specific genetic signals and selection patterns captured by our framework, we focus the detailed variant-level analysis, including Manhattan and UpSet plots, on blood glucose as the representative phenotype. Figure 2 (top panel) displays overlaid Manhattan plots derived from QR-GWAS across quantile levels $\tau = 0.1, 0.2, \ldots , 0.9.$ Compared to conventional linear regression GWAS (bottom panel), QR-GWAS detects a broader spectrum of significant variants, including numerous loci that linear regression fails to capture. Although individual quantiles may yield fewer signals in isolation compared to linear regression, aggregating results across quantiles unveils variants whose effects are otherwise obscured by heterogeneity or non-normality.

**Fig. 3:**
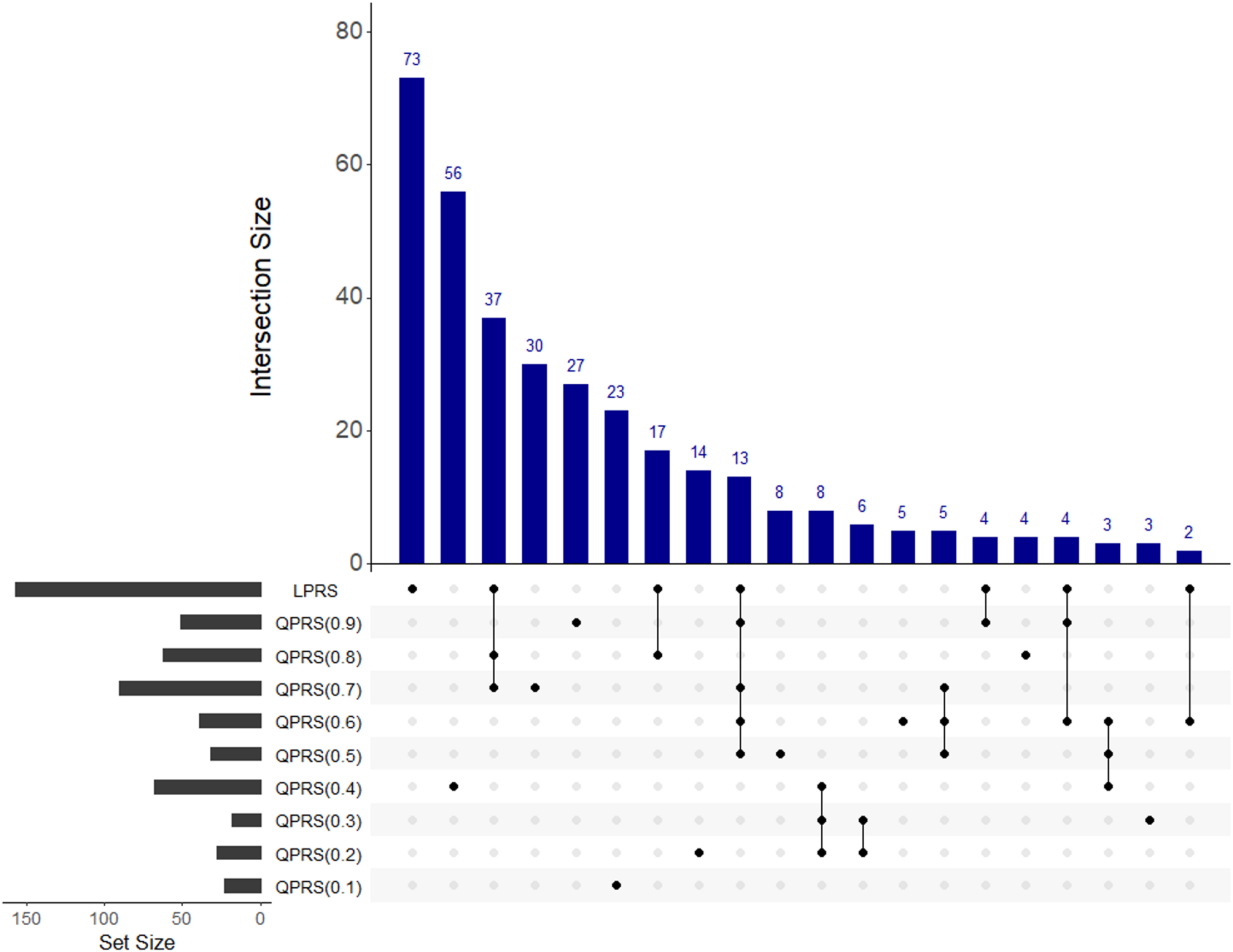
Upset plot of SNP overlaps among LPRS and QPRS from ${ tau } = 0.1$ to 0.9. The horizontal bars (set size) show the total number of SNPs with *p*-values below $1 \times 10^{ - 5}$ identified by each PRS. In the dot matrix below, filled circles indicate the PRSs involved in each intersection, and the vertical bars (intersection size) represent the number of SNPs shared across those models.

Figure 3 presents an UpSet plot illustrating overlaps among significant SNPs detected by LPRS and QPRS. The horizontal bars display the total number of SNPs identified by each method, while vertical bars show intersections across methods. In the dot matrix below, filled circles indicate the sets included in each intersection, with connecting lines denoting the specific combinations compared. QPRS identifies 342 significant SNPs, of which 77 overlap with LPRS, while 73 are unique to LPRS and 265 are unique to QPRS. Notably, 56 SNPs are uniquely identified at QPRS(0.4), while additional unique discoveries also appear at other quantiles, particularly QPRS(0.1) (23 SNPs) and QPRS(0.9) (27 SNPs). The concentration of discoveries at $\tau = 0.4$ is likely attributable to the right-skewed nature of the 2-h glucose distribution in our data. In such a distribution, the mode, the point of highest data density, is typically located to the left of the median ($\tau = 0.5$). Consequently, the $\tau = 0.4$ quantile falls within this high-density region. Although tail quantiles tend to have lower power, they can reveal distribution-specific effects. The discoveries at QPRS(0.1) and QPRS(0.9) suggest that certain genetic effects are more apparent in the lower or upper tails of the phenotype distribution.

We highlight rs7306855 and rs17153083 as illustrative examples of SNPs exhibiting heterogeneous effects on 2-h post-OGTT blood glucose levels. The effect sizes of these variants vary substantially across the phenotype distribution, a pattern effectively masked by conventional mean-based estimates. Specifically, for SNP rs7306855, the effect is strongly positive at lower quantiles ($\hat{\beta } = 6.72$ at $\tau = 0.1$) , indicating an upward shift in the lower conditional quantiles of glucose given genotype. However, this effect diminishes and sharply reverses, becoming strongly negative in the upper tail ($\hat{\beta } = - 15.00$ at $\tau = 0.9$). Similarly, SNP rs17153083 exhibits a strongly negative effect at the highest quantile ($\hat{\beta } = - 10.22$ at $\tau = 0.9$). In both cases, the mean regression provides a single, uninformative estimate ($\hat{\beta } = - 0.44$ and $0.05$, respectively) that fails to capture these complex relationships. These patterns are representative examples of heterogeneous effects. The signals from these SNPs are largely obscured in a mean regression but are clearly revealed when modeling quantile-specific effects. These examples, therefore, illustrate the type of complex genetic signal that motivates the QPRS framework; they highlight that QPRS can capture heterogeneous genetic influences that LPRS methods are not designed to detect.

We next assess the predictive performance of QPRS relative to five benchmark methods: LPRS, Lassosum, LDpred, SBLUP, and PRS-CS. As outlined in “Materials and methods” section, performance is evaluated under two frameworks: (a) PRS-only models and (b) covariate-adjusted models (controlling for sex, age, BMI, smoking status, and 10 principal components). Table 5 reports the results from fivefold cross-validation. For ease of comparison, all $R^{2}$ values reported in Table 5 and discussed below are multiplied by 10.

**Table 5:** Predictive performance of polygenic risk score methods under PRS-only and covariate-adjusted models (adjusted for sex, age, BMI, smoking status, 10 PCs).

Phenotype	PRS-only				Covariates-adjusted			
	TG	GLU	GLU_bin_		TG	GLU	GLU_bin_	
Method	$R^{2}$	$R^{2}$	AUC	AUCL	$R^{2}$	$R^{2}$	AUC	AUCL
LPRS	0.008 (0.014)	− 0.111 (0.043)	0.515 (0.009)	0.505 (0.003)	0.253 (0.007)	0.040 (0.021)	0.555 (0.038)	0.521 (0.014)
Lassosum	0.344 (0.036)	− 0.082 (0.030)	0.516 (0.013)	0.506 (0.005)	0.607 (0.022)	0.044 (0.028)	0.552 (0.037)	0.520 (0.014)
LDpred	0.223 (0.031)	− 0.095 (0.027)	0.515 (0.015)	0.505 (0.005)	0.481 (0.024)	0.028 (0.030)	0.579 (0.038)	0.530 (0.014)
SBLUP	0.008 (0.022)	− 0.099 (0.035)	0.510 (0.010)	0.504 (0.004)	0.253 (0.021)	0.051 (0.028)	0.555 (0.034)	0.521 (0.013)
PRS-CS	0.114 (0.024)	− 0.105 (0.037)	0.521 (0.013)	0.508 (0.005)	0.369 (0.029)	0.047 (0.026)	0.545 (0.036)	0.517 (0.013)
QPRS(0.5)	0.047 (0.025)	− 0.091 (0.033)	0.500 (0.000)	0.500 (0.000)	0.303 (0.049)	0.024 (0.030)	0.572 (0.037)	0.527 (0.014)
QPRS	0.344 (0.053)	− 0.084 (0.029)	0.520 (0.009)	0.507 (0.003)	0.624 (0.048)	0.059 (0.026)	0.560 (0.033)	0.533 (0.012)
LPRS + QPRS	0.350 (0.053)	− 0.107 (0.036)	0.504 (0.008)	0.501 (0.003)	0.623 (0.047)	0.067 (0.019)	0.564 (0.038)	0.531 (0.014)

AUCL denotes liability AUC. Standard errors from fivefold CV are reported in parentheses. GLU denotes 2-h post-OGTT blood glucose levels, and GLU_bin_ denotes glucose status (> 200 mg/dL vs. ≤ 200 mg/dL). All $R^{2}$ values are multiplied by 10.

We first examine predictive performance for TG. In the PRS-only framework, the combined model (LPRS + QPRS) attains the highest out-of-sample R^2^ of 0.350, with QPRS and Lassosum each yielding 0.344, both substantially exceeding all other competing methods, with LPRS lagging markedly at 0.008. Under the covariate-adjusted framework, QPRS again achieves the highest $R^{2}$ of 0.624, with the combined model performing comparably at 0.623, both substantially exceeding LPRS (0.253) and all other benchmarks. These results demonstrate that QPRS captures distributional genetic signals undetectable by conventional mean-based estimators, with the most pronounced gains observed for a right-skewed phenotype whose genetic architecture exhibits pronounced heterogeneity across the outcome distribution.

For 2-h post-OGTT glucose, out-of-sample $R^{2}$ values are uniformly low across all methods under both frameworks, reflecting the inherently limited marginal polygenic signal for this trait after accounting for clinical covariates. Lassosum and QPRS attain the least negative values, whereas LPRS records the most severe deficit at − 0.111. Under the covariate-adjusted framework, all methods recover positive $R^{2}$, with the combined model attaining the highest value of 0.067, followed by QPRS at 0.059. For the binary diabetes outcome under the covariate-adjusted framework, LDpred achieves the highest observed-scale AUC of 0.579; however, on the liability scale, which adjusts for population prevalence and is less susceptible to ascertainment bias, QPRS attains the highest AUCL of 0.533, followed by the combined model at 0.531 and LDpred at 0.530. Taken together, these results demonstrate that QPRS consistently enhances polygenic prediction by incorporating distributional genetic information beyond the conditional mean, with particularly salient gains for right-skewed metabolic traits such as TG.

To rigorously assess the incremental value of the proposed method, we evaluate the statistical significance of predictive gains using paired $t$-tests across the five independent test folds. For TG, QPRS and the combined model demonstrate statistically significant improvements over LPRS, SBLUP, PRS-CS, and LDpred (*p* < 0.05), while yielding predictive performance statistically comparable to Lassosum. For 2-h post-OGTT glucose, the uniformly low polygenic signal limits statistical power to detect meaningful differences across methods; nonetheless, the combined model consistently attains the highest out-of-sample $R^{2}$ and liability-scale AUC among all competing approaches.

**Fig. 4:**
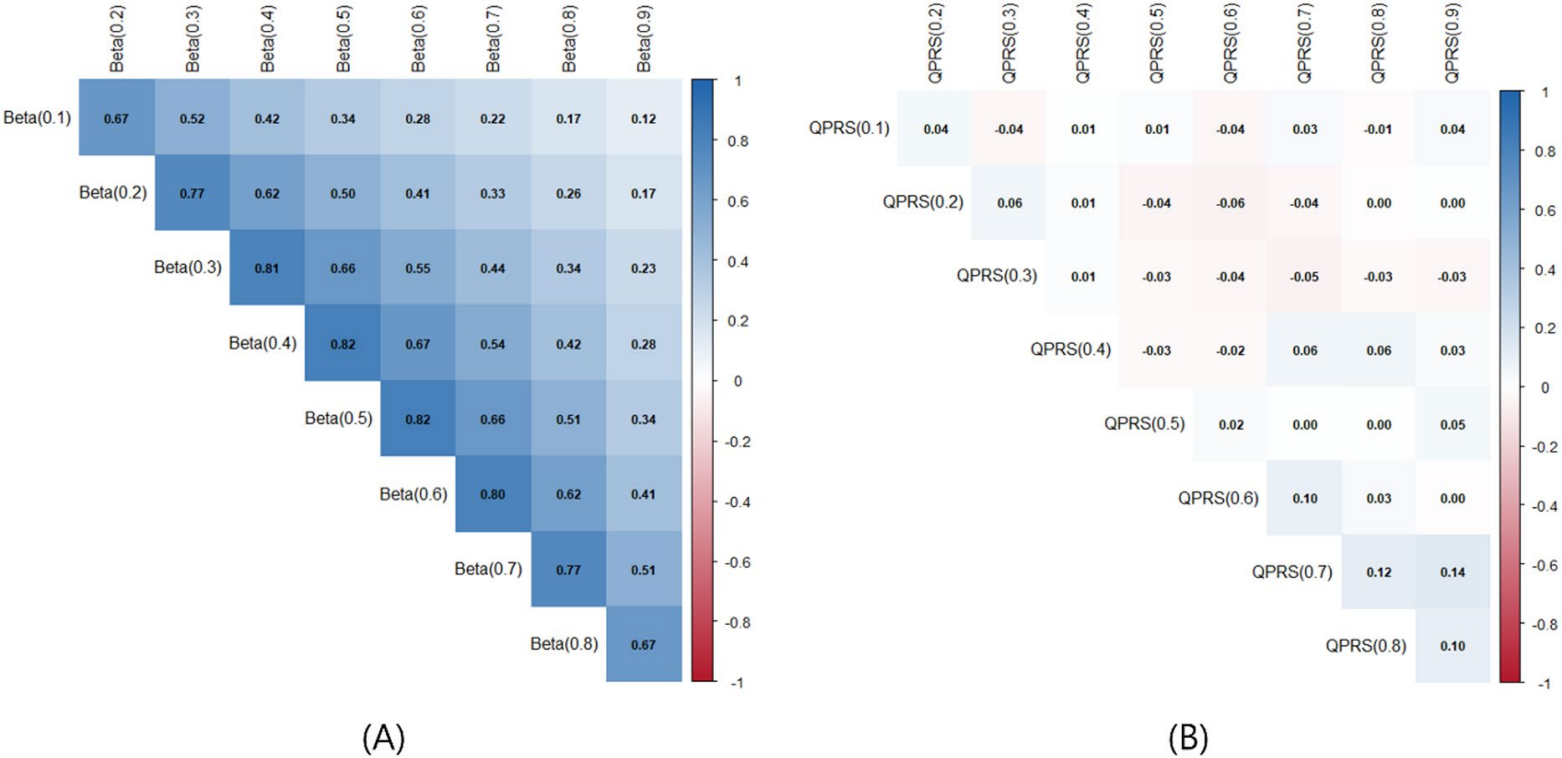
Pairwise correlation structures of quantile-specific genetic components. (**A**) Pearson correlation matrix of estimated SNP effect sizes across quantiles ($\tau = 0.1$ to 0.9). (**B**) Pearson correlation matrix of the computed QPRS($\tau$) values.

Next, to show the relationships among quantile-specific components, we analyze the pairwise correlations of both the estimated SNP effects, $\hat{\beta }\left( \tau \right)$, and the resulting individual risk scores QPRS($\tau$). As illustrated in Fig. 4A, the effect estimates exhibit strong positive correlations between adjacent quantiles, reflecting the biological continuity of genetic effects across the phenotype distribution. Conversely, Fig. 4B reveals that the derived individual risk scores exhibit minimal correlation. This independence is largely attributable to the discrete nature of the C + T procedure, which selects distinct subsets of tail-specific variants for each quantile. From a modeling perspective, this low correlation is advantageous; it implies that the joint model aggregates complementary, non-redundant genetic signals without suffering from multicollinearity, thereby maximizing predictive efficiency.

## Discussion

This study highlights the importance of modeling heterogeneity in genetic effects when constructing PRSs. By incorporating QR into the PRS framework, we show that genetic influences can vary systematically across the phenotype distribution, with implications for both prediction and discovery. The observed gains in predictive accuracy, particularly in the presence of skewed phenotypes such as TG, illustrate that mean-based models may underestimate genetic contributions in distributional extremes.

The ability of QR-GWAS to identify variants missed by linear models further emphasizes the biological relevance of distribution-specific effects. These findings align with emerging evidence that genetic associations may manifest through interactions with phenotypic scale, environment, or unmeasured modifiers, resulting in non-uniform effects across quantiles. In this light, QPRS does not merely refine prediction, but also provides an additional lens for exploring the architecture of complex traits. Our empirical results confirm the theoretical expectation that joint modeling reduces prediction variance. As evidenced by the superior performance of the combined model over the single-quantile models (e.g., QPRS(0.5)) in simulations and in the analysis of TG, integrating adjacent quantile signals effectively compensates for the noise in tail-specific estimates.

Our approach complements existing PRS strategies rather than replacing them. The consistent improvement when QPRS is combined with conventional linear PRS suggests that distinct layers of information are captured by mean- and quantile-based modeling. This complementarity may prove particularly valuable in precision medicine, where capturing variability across risk strata is crucial for effective stratification.

A theoretical consideration in estimating quantile-specific effects is the potential for quantile crossing, where the estimated quantile functions violate the monotonicity property of the conditional distribution. While our current QPRS framework utilizes these scores primarily as joint covariates, ensuring logical ordering can further enhance the interpretability of individual quantile effects. To address this, one effective post-processing approach is the rearrangement method^39^, which involves sorting the estimated quantiles to restore monotonicity. For future development, the QPRS framework could be extended to incorporate monotone constrained optimization. By estimating multiple quantiles simultaneously under non-crossing constraints, such a joint estimation approach would strictly enforce structural validity across the entire distribution.

Several extensions naturally follow. Penalized^40^ or Bayesian^41^ QR could be incorporated to improve SNP selection beyond clumping and thresholding. Developing a Lassosum-like approach to leverage large-scale QR-GWAS summary statistics is an important direction for future work. Additionally, future research could explore inferring individual-specific risk strata based on genetic profiles or covariates to construct personalized QPRS, thereby bypassing the circularity of relying on observed phenotypes. Moreover, validation across multiple traits and ancestries will be necessary to establish the generalizability of QPRS.

## Supplementary Information

Below is the link to the electronic supplementary material.

Supplementary Material 1

## Data Availability

Genotype data from the Korea Association Resource (KARE) cohort, which is part of the Korean Genome and Epidemiology Study (KoGES), were accessed through the Clinical and Omics Data Archive (CODA; https://coda.nih.go.kr) of the Korea Disease Control and Prevention Agency (KDCA).
