# Supplementary material for: Enhancing polygenic risk prediction by modeling quantile-specific genetic effects
Source: Sci Rep. 2026 Apr 6;16:11749. doi: 10.1038/s41598-026-47082-9 (PMC13061970; doi:10.1038/s41598-026-47082-9)
Supplement: Supplementary file 1 — Supplementary Material 1 [file 41598_2026_47082_MOESM1_ESM.pdf]

Supplementary materials for Enhancing Polygenic Risk Prediction by Modeling Quantile-Specific Genetic Effects

1. Characteristics of the Study Population

The Korea Association Resource (KARE) cohort is part of the Korean Genome and Epidemiology Study (KoGES), a large-scale community-based prospective cohort study initiated by the Korea National Institute of Health (KNIH). The cohort consists of community-dwelling residents aged 40–69 years recruited from the urban area of Ansan and the rural area of Ansung in the Republic of Korea. Baseline examinations were conducted between 2001 and 2002, comprising extensive epidemiological surveys, physical examinations, and biospecimen collection. Table S1 summarizes the baseline demographic and clinical characteristics of the participants (n = 8408) included in our study.

**Characteristics**	Diabetes status		Total (N = 8,408)
	Control	Case	
Age, years	51.72 ± 8.82	54.78 ± 8.95	51.93 ± 8.86
Sex, female, n (%)	3642 (46.56%)	312 (53.33%)	3954 (47.02%)
Body Mass Index (BMI), kg/m²	24.48 ± 3.09	24.58 ± 3.45	24.56 ± 3.13
Smoking status, n (%)			
Non-smoker	4601 (55.45%)	302 (3.64%)	4903 (59.09%)
Ex-smoker	1168 (14.08%)	99 (1.19%)	1267 (15.27%)
Current smoker	1951 (23.51%)	176 (2.11%)	2127 (25.64%)
**Diabetes-related Traits**			
Fasting Glucose	83.43 ± 10.20	124.46 ± 44.28	86.30 ± 18.90
2-h Post-load Glucose, mg/dL	116.29 ± 31.10	261.63 ± 62.85	126.09 ± 49.96
HbA1c (%)	5.58 ± 0.40	7.18 ± 1.59	5.69 ± 0.71
**Lipid-related Traits**			
Total Cholesterol, mg/dL	177.61 ± 141.61	175.22 ± 139.79	177.44 ± 141.47
Triglycerides, mg/dL	147.42 ± 102.64	204.67 ± 154.83	151.33 ± 107.82
HDL Cholesterol, mg/dL	44.94 ± 10.15	43.65 ± 9.87	44.84 ± 10.12

Table S1. Characteristics of the KARE cohort. Diabetes status was defined based on fasting plasma glucose ≥126 mg/dL and/or 2-hour post-load glucose ≥200 mg/dL during the oral glucose tolerance test (OGTT). Data are presented as mean ± standard deviation (SD) for continuous variables and number (%) for categorical variables.

2. Additional results for simulation scheme 1

To address the concern that simulation scheme 1 may favor variance effects and thus systematically advantage QPRS, we conduct an additional simulation under a mean-dominant regime. In this setting,

both the mean-effect size and the variance-effect size were set to 1, with $p_1 = 10$ causal mean-effect variants and $p_2 = 5$ variance-effect variants. Compared to the main simulations, this parameterization reduces the relative contribution of variance modulation and better reflects scenarios in which mean-based genetic effects remain primary, while quantile-specific effects are present but modest.

Table S1 reports the mean squared error (MSE) for estimating conditional quantiles at $\tau = 0.1, \ldots, 0.9$ under three error distributions. Across all distributions, LPRS and QPRS show comparable performance at the median quantile ($\tau = 0.5$), indicating that mean-based signals dominate prediction at the center of the phenotype distribution. At the lower and upper tails ($\tau = 0.1$ and $\tau = 0.9$), QPRS consistently achieves lower MSE than LPRS, although the magnitude of improvement is moderate compared to the main simulation settings. This pattern is particularly evident under heavy-tailed and skewed error distributions, where quantile-specific effects are more pronounced. The single-quantile approach QPRS(τ) performs best at its corresponding target quantile but does not provide uniform improvements across the distribution. Overall, these results demonstrate that when variance effects are attenuated, QPRS does not overwhelm mean-based PRS but instead yields stable, incremental gains at non-central quantiles, consistent with the empirical pattern observed in real-data analysis.

Table S2 summarizes SNP selection performance using precision, recall, and the size of the selected SNP set. Under this mean-dominant regime, LPRS attains higher precision in some settings, reflecting its focus on central mean effects, whereas QPRS continues to achieve substantially higher recall across all error distributions. This indicates that QPRS remains more sensitive to heterogeneous signals, even when such effects are weak. Compared to the main simulation results, the difference in the size of the selected SNP sets between LPRS and QPRS is reduced, suggesting that the dominance of variance effects is no longer driving large discrepancies in model complexity.

Taken together, these findings confirm that the advantage of QPRS under simulation scheme 1 is not an artifact of overly strong variance effects. Instead, QPRS provides consistent but moderate improvements over LPRS in settings where mean effects dominate, while still offering enhanced sensitivity to quantile-specific genetic contributions. This behavior closely mirrors the performance observed in real-data applications and supports the robustness of the proposed framework across a range of genetic architectures.

τ	$\epsilon \sim N(0,1)$			$\epsilon \sim t(5)$			$\epsilon \sim logN(0,1)$		
	LPRS	QPRS	QPRS(τ)	LPRS	QPRS	QPRS(τ)	LPRS	QPRS	QPRS(τ)
0.1	13.10<br>(0.54)	10.59<br>(0.56)	13.16<br>(0.47)	16.14<br>(0.64)	12.39<br>(0.65)	15.37<br>(0.61)	14.70<br>(0.52)	11.97<br>(0.66)	15.20<br>(0.78)
0.2	7.80<br>(0.52)	6.49<br>(0.48)	8.08<br>(0.48)	8.00<br>(0.45)	6.43<br>(0.49)	8.27<br>(0.54)	11.37<br>(0.41)	9.38<br>(0.47)	11.64<br>(0.52)
0.3	6.27<br>(0.57)	5.55<br>(0.50)	6.13<br>(0.55)	5.83<br>(0.54)	5.26<br>(0.52)	5.91<br>(0.53)	8.77<br>(0.34)	7.35<br>(0.39)	8.94<br>(0.36)
0.4	6.58<br>(0.61)	6.17<br>(0.55)	6.49<br>(0.59)	6.09<br>(0.58)	5.91<br>(0.54)	6.05<br>(0.56)	6.51<br>(0.29)	5.71<br>(0.34)	6.52<br>(0.33)
0.5	8.05<br>(0.65)	7.94<br>(0.60)	8.08<br>(0.61)	7.98<br>(0.62)	7.78<br>(0.58)	7.94<br>(0.61)	4.71<br>(0.31)	4.47<br>(0.35)	4.82<br>(0.30)

0.6	10.71<br>(0.70)	10.90<br>(0.75)	10.92<br>(0.73)	11.02<br>(0.74)	10.89<br>(0.75)	11.08<br>(0.73)	4.01<br>(0.38)	4.36<br>(0.41)	3.91<br>(0.36)
0.7	14.88<br>(0.95)	15.13<br>(0.94)	14.72<br>(0.96)	15.91<br>(1.08)	15.68<br>(1.05)	15.72<br>(1.09)	6.55<br>(0.52)	7.04<br>(0.53)	6.27<br>(0.49)
0.8	20.98<br>(1.39)	21.00<br>(1.34)	20.17<br>(1.44)	23.71<br>(1.63)	22.99<br>(1.59)	22.12<br>(1.52)	18.20<br>(1.20)	18.05<br>(1.31)	16.97<br>(1.11)
0.9	30.87<br>(2.19)	31.48<br>(2.29)	29.78<br>(2.08)	38.91<br>(2.73)	38.01<br>(2.74)	36.62<br>(2.62)	65.86<br>(4.36)	61.82<br>(4.71)	61.39<br>(4.29)

Table S2. Mean squared error (MSE) for estimating conditional quantiles at $\tau = 0.1, \dots, 0.9$ with $p_1 = 10$ mean-effect variants and $p_2 = 5$ variance-effect variants. Results are shown for LPRS, QPRS, and single-quantile QPRS(τ) across normal, heavy-tailed, and skewed error distributions. Standard errors are reported in parentheses.

Score	$\epsilon \sim N(0,1)$			$\epsilon \sim t(5)$			$\epsilon \sim logN(0,1)$		
	Precision	Recall	$\|S\|$	Precision	Recall	$\|S\|$	Precision	Recall	$\|S\|$
LPRS	0.51<br>(0.04)	0.45<br>(0.03)	17.84<br>(2.67)	0.19<br>(0.01)	0.87<br>(0.02)	90.40<br>(21.03)	0.44<br>(0.03)	0.79<br>(0.02)	31.88<br>(4.22)
QPRS	0.22<br>(0.02)	0.55<br>(0.04)	140.84<br>(54.82)	0.32<br>(0.02)	0.75<br>(0.06)	37.92<br>(3.82)	0.39<br>(0.02)	0.52<br>(0.04)	22.20<br>(2.44)
QPRS(0.5)	0.35<br>(0.02)	0.57<br>(0.02)	28.48<br>(2.63)	0.30<br>(0.02)	0.83<br>(0.02)	47.20<br>(4.22)	0.44<br>(0.03)	0.55<br>(0.02)	21.64<br>(1.85)

Table S3. SNP selection performance under the simulation setting with $p_1 = 10, p_2 = 5$, mean-effect size = 1, variance-effect size = 1. Precision, recall, and the size of the selected SNP set ($|S|$) are reported for LPRS, QPRS, and QPRS(0.5) across different error distributions. Standard errors are shown in parentheses.
